# Sensory Hyper‐Sensitivity Associates With Poor Infant Sleep and Different Parental Sleep Practices, in a UK Cohort

**DOI:** 10.1111/infa.70129

**Published:** 2026-10-07

**Authors:** Teodora Gliga, Hope Fincham, Anna De Laet, Rachael Bedford

**Affiliations:** ^1^ School of Psychology University of East Anglia Norwich UK; ^2^ Department of Psychology Centre for Brain and Behavior Queen Mary University of London London UK

## Abstract

Sleep difficulties (e.g., increased sleep onset latency and more frequent night awakenings) are common in early development and are associated with increased parental stress. Sleep difficulties have been linked to sensory hypersensitivity in typically developing infants and infants with family history of autism. In the current study, we investigate whether infant sensory sensitivity is associated with the strategies parents employ when settling infants to sleep. Strategies encouraging autonomy, rather than those involving active physical comforting, have previously been shown to lead to better sleep. We report findings from a cross‐sectional UK sample of 245 infants aged 6–13 months, from which we collected online parent‐report measures of infant sleep and sensory sensitivity (ITSP). We replicate previous findings showing that poorer sleep is correlated with higher infant sensory sensitivity. We then show an association between higher infant sensory sensitivity and more frequent use of all settling strategies, as well as a higher number of different strategies used. A strong correlation between infant and maternal sensory sensitivity precluded testing whether parental settling strategies are driven by infant or parental sensory sensitivity. The current findings emphasize the need to integrate information about intrinsic (e.g., infant sensitivity) and social environment factors (e.g., parental sensitivity, settling strategies) to understand infant sleep.

## 1 Introduction

The past two decades have seen robust evidence emerge in support of an active role of sleep in knowledge consolidation (Rasch and Born 2013; Staresina 2024), including in development (Dionne et al. 2011; Huber and Born 2014; Mason and Spencer 2022). These discoveries have fueled an increasing interest in understanding the sources of individual variation in sleep and the impact this variation may have on brain development and learning. Sleep changes dramatically in the first year of life, with a gradual establishment of a day and night rhythm (Iglowstein et al. 2003; Blair et al. 2012; Galland et al. 2012). In parallel with a decrease in daytime sleep, infants also get better at falling asleep and staying asleep (Paavonen et al. 2020). In a recent UK longitudinal study, parents reported that sleep onset latencies, a measure of how long it takes infant to settle to sleep, decrease from 11 minutes (on average) at 5 months‐of‐age to 8 minutes at 10 months and 6 minutes at 14 months (De Laet et al. 2022). The number of times parents were aware of infants waking up at night also decreased from 2.4 to 1.2 to 1 time per night, over the same time frame.

In addition to these normative changes, there is also important individual variation. For example, in the above study, which was part of the British Autism Study of Infant Siblings (BASIS), a cohort of 65 infants with an increased familial likelihood of developing autism (because they had an older autistic sibling), did not show this same developmental decrease in sleep onset latencies and awakenings (De Laet et al. 2022). In the same cohort, lower sleep quality was associated with lower later cognitive development and social functioning (Begum‐Ali et al. 2023). Given what we know about the role of sleep in driving brain and cognitive development (Lokhandwala and Spencer 2022), identifying factors that might affect sleep in infancy is critical for understanding developmental pathways.

One factor that has been repeatedly shown to be associated with poor sleep is sensory sensitivity. This association is accentuated in samples with neurodevelopmental conditions, such as autism, but is also present in the general population. Appleyard et al. (2020) found poor sleep and higher sensory sensitivity to be concurrently associated in a group of 160 typically developing 2.5‐year‐olds. Higher sensory sensitivity was associated with longer sleep onset times in a group of 177 typically developing infants, aged 2–36 months (Vasak et al. 2015). In a study of 69 autistic and 62 non‐autistic 3‐ to 7‐year‐olds, longer sleep onset, more parasomnias, and overall poor sleep scores were associated with tactile hypersensitivity (Tzischinsky et al. 2018). This association is present also when objective measures of sensory processing are used. In the BASIS cohort, longer sleep onset times, measured by parental report, were associated with a neural measure of sensory over‐sensitivity, that is, an attenuation in Event Related Potential amplitude to repeated tactile stimuli. Infants that had smaller attenuation, took longer to fall asleep (De Laet et al. 2022). Thus, there is now strong converging evidence that sensory sensitivity is associated with poor sleep across typical and atypical development, and suggestive evidence that this link emerges early, in the first year of life.

According to a transactional model of infant sleep development (Sadeh and Anders 1993) sleep is the product of intrinsic factors (such as neurodevelopmental stage, health, and temperament) and their dynamic relationships with extrinsic factors (such as cultural norms, socioeconomic factors, and family environment). While infant sensory sensitivity may directly interfere with infant settling, a rich literature suggests that parental care, in the form of the settling strategies parents employ, plays an important role helping infants fall asleep. The Parental Interactive Bedtime Behavior Scale (PIBBS, Morrell and Cortina‐Borja 2002) is a questionnaire widely used to capture the breadth of parental settling strategies. Strategies are classified based on how involved the parent is with the infant; *active strategies* include rocking or carrying the child in arms, while strategies that *encourage autonomy* include standing next to the child's bed, giving them a toy to hold or leaving them to settle on their own. The use of active strategies tends to decrease with age, while the use of strategies encouraging autonomy increases (Morrell and Cortina‐Borja 2002). Studies using this measure found that infants whose parents more frequently used strategies encouraging autonomy had fewer sleep problems, both concurrently (Morrell and Steele 2003; Sadeh et al. 2009; Schlarb et al. 2020) and later in toddlerhood (Morrell and Steele 2003), and childhood (Sheridan et al. 2013; Cowie et al. 2016). Strategies that encourage autonomy were shown to be associated with infants using more self‐regulatory behaviors such as soothing themselves back to sleep (France et al. 2003; Adams et al. 2022; Leerkes et al. 2025).

There is mixed evidence on whether and which child and parental characteristics influence the type of settling strategies parents employ. For example, maternal involvement in infant settling was greater in mothers experiencing a higher level of psychosocial adversity (Cronin et al. 2008) and predicted less optimal sleep at five years (Sheridan et al. 2013). In their original study, Morrell and Cortina‐Borja (2002) described an association between increased maternal cognitions about sleep (e.g., more doubts about parenting competence, anger at child's demands during the night) and more frequent use of active physical comforting over encouraging autonomy. No associations were found with infant temperament (e.g., the child being fussy or difficult). Understanding the factors that drive sleep settling strategies is important to gain a complete picture of sleep in early development.

In this study, we are interested in understanding the impact of infant sensory sensitivity on the strategies that parents employ to settle infants to sleep. For example, active physical settling strategies are rich in multi‐modal stimulation (e.g., touch and smells when carrying in arms or feeding to sleep), which more sensitive infants may experience differently; parents of infants with higher sensory sensitivity may use active physical settling less frequently, instead opting for strategies encouraging autonomy. Alternatively, hypersensitive infants' difficulty with falling asleep may increase parental anxiety and lead to more active involvement in settling. We test whether infant sensory hypersensitivity is associated with the use of different types of parental settling strategies, in the first year of life. Sadeh et al. (2009) showed that, from around 3–5 months, parents start using sleep routines, with about half of infants being moved in their own room (Sadeh et al. 2009). Infants included in our cross‐sectional sample are therefore older than 6 months and up to 13 months. To increase the proportion of infants and parents with sensory hypersensitivity, we also recruited infants with an older autistic sibling (17% of the sample), which was ascertained based on parental reports. Given the correlation between parent and infant sensory sensitivity (Glod et al. 2017), parent sensory sensitivity itself may also influence settling strategies, with potential cumulative effects on infant sleep. Data on parental sensitivity was therefore also collected.

## 2 Methods

### 2.1 Participants

Parents were recruited via advertising on social media and through mailing lists. We aimed to recruit 300 participants, a sample similar to that recruited by Morrell and Cortina‐Borja (2002). Data collection was stopped once 350 entries were recorded. Participants with fewer than 10% of questionnaires completed were removed (*N* = 74). Infants outside the age range required in the current analysis (aged 6–13 months) were removed (*N* = 31). A final sample of 245 infants, with a mean age of 8.16 months, was included in the analysis (see Table 1 for demographics). Family income had a representative distribution for UK (Supporting Information S1: Figure S1). Forty‐seven participants had an older autistic sibling, which was ascertained based on parental report. Given the challenges of using parental report to indicate a diagnosis of autism (but see Bazelmans et al. 2024), we did not use this as a factor in analysis. However, we provide evidence in supplementary material that infants whose parents reported familial autism had higher sensory sensitivity, suggesting that targeting recruitment to this population yields a higher variability of traits (Supporting Information S1: Table S1 and Figure S3).

**TABLE 1:** Descriptives and main variables of interest.

	Age (months)	Sex (Boy: Girl)	SOL (min)	Awakenings	Infant sensory sensitivity	Maternal sensory sensitivity
Mean	8.16	138:107	22.28	2.73	2.31	64.90
Range	6–13		0–77.5	0–14	0–4.16	3–123
N	245	245	245	245	245	226

Abbreviation: SOL = Sleep Onset Latency.

### 2.2 Design and Procedure

Parents (96% mothers) completed a series of online questionnaires, using Qualtrics platform, which they accessed following a hyperlink posted on social media or in an email sent to a parent database held at University of East Anglia. To prevent multiple entries from the same participant or entries from bots, we asked participants to create a unique identifier made of the child initials and their date of birth (e.g., AB12082017). No duplicates were detected.

### 2.3 Measures

#### 2.3.1 Settling Strategy Questionnaire

The settling strategies used by parents were investigated using the Parental Interactive Bedtime Behavior Scale (PIBBS, Morrell and Cortina‐Borja 2002). This questionnaire asks parents to rate how often they employ 21 settling behaviors, using a five‐point Likert scale that ranges from never (scoring zero) to very often (scoring four). The authors of the questionnaire reported a Cronbach's alpha of 0.7, indicating good internal consistency. To reduce dimensionality we grouped individual questions, by averaging, within 5 factors. The grouping was guided by Morrell and Cortina‐Borja’s (2002) factor analysis of data from a sample of infants aged 12–19 months (mean 13.77) and by conceptual scrutiny of the items. Our final 5 categories were created by averaging items withing the following Settling strategy types: Active physical comforting (average of Stroking, Cuddling or rocking in arms, Carrying in arms, Feed or give drink, Settle in parent's bed, Settle on sofa with parent), Settle by movement (Walks in pram, Car rides), Social comforting (Sing lullaby, Talking softly to child, Reading a story, Play, Stand near cot, Lie next to baby), Encourage autonomy (Offer special toy/blanket, Dummy, Musical toy, White or other noise, Leave to cry, Settle on their own), and Giving medicine, which was an item on its own. There are two differences with Morrell & Cortina‐Borja in that (1) we kept the Giving medicine item in the analyses (which they discarded because of very low ratings) and (2) we grouped their Passive Physical comforting and Social comforting scales into one Social Comforting scale since we deemed the items to be conceptually close. Other studies have used a similar 4 factors structure (Cronin et al. 2008). The PIBBS measure of sleep settling conflates the frequency of use for each settling strategy and the number of different strategies used. We therefore also calculated a *Settle variability* measure by counting settling strategies that were ever used (i.e. had a frequency score > 0).

#### 2.3.2 Infant Sensory Sensitivity

Infant sensory sensitivity was measured using the Infant Toddler Sensory Profile 2 (ITSP, Dunn 2014). The ITSP2 is normed for infants 7‐ to 35‐months‐of‐age and has been used previously with parents of infants in our age range (McGeorge et al. 2015; Tauman et al. 2017) including infants with an older autistic sibling (Bussu et al. 2025). The 54‐item questionnaire assesses general, auditory, visual, tactile, vestibular, and oral sensory processing and the behavioral responses associated with sensory processing. Questions ask how the infant responds to sensory input during daily life, using statements such as, “my child only pays attention if I speak loudly.” The parent rates the frequency of those behaviors on a six‐point Likert scale, with the options: almost always (scored as 5), frequently, half the time, occasionally, almost never, or does not apply (scored as zero). Questions in each sensory modality reflect different sensory processing patterns and are categorized according to the quadrants of Dunn's Model of Sensory Processing (Dunn and Daniels 2002); “low registration” reflects a high neurological threshold and passive self‐regulation strategies, “sensation seeking” reflects a high neurological threshold and active self‐regulation strategies, “sensory sensitivity” reflects a low neurological threshold and passive self‐regulation strategies and “sensation avoiding” reflects a low neurological threshold and active self‐regulation strategies (Dunn and Daniels 2002). The authors of the questionnaire reported convergent and discriminate validity, and the Cronbach's alpha coefficient measure of internal consistency is 0.70–0.86. As suggested by Dunn and Daniels, a *Low Threshold* score was generated using the average of the “sensation avoiding” and “sensory sensitivity” quadrants, (2002). The higher the Low Threshold score, the more sensitive the infant. We will refer to this measure as the infant sensory sensitivity.

#### 2.3.3 Infant Sleep

The questionnaire asked parents to consider their infant's sleep pattern over the past week and was built based on questions used in many other infant sleep questionnaires (e.g., the Sleep and Settle Questionnaire; Matthey 2001). Parents reported the average number and duration of daytime naps, how long on average the infant took to settle for the night and during the day, the average wake time in the morning, the average time for falling asleep at night, the average number of night awakenings and how long they lasted. We did not ask about settling times when infants woke up during the night. Two continuous sleep variables were used in analysis: sleep onset latency (SOL, the average of the infant's settling time durations for daytime naps and for the night‐time sleep), and number of awakenings, since these have been shown to relate to infant sensory sensitivity in previous work (De Laet et al. 2022). One outlier value likely resulted from participant input error and was excluded from the SOL data (value of 250 minutes given for sleep onset latency at night).

#### 2.3.4 Maternal Sensory Sensitivity

Parental sensory sensitivity was assessed using the Glasgow Sensory Questionnaire (GSQ; Robertson and Simmons 2013). The GSQ has 42 questions to assess sensory sensitivity in the visual, auditory, proprioceptive, gustatory, vestibular, olfactory, and tactile domains, with questions asking both about hypo‐ and hyper‐sensitivity for example “Do you notice that you have hurt yourself but did not feel any pain” (tactile, hypo‐sensitivity) or “Do you react very strongly when you hear an unexpected noise” (auditory, hyper‐sensitivity). Responses are given on five‐point Likert scales from never (scoring zero) to always (scoring four). The GSQ variable is a sum of all the responses to the Glasgow Sensory Questionnaire (Robertson and Simmons 2013). Total scores on the questionnaire range from 0 to 168, with higher scores denoting increased sensory atypicality. The GSQ has a Cronbach's alpha of 0.935 and the authors reported reasonable face and content validity. This questionnaire was added later to the study, therefore only 228 (82%) of participants contributed these data. We will refer to this measure as the maternal sensory sensitivity.

### 2.4 Analysis Plan

We start by characterizing the sample, using bivariate associations between key measures: infant sleep, parental and infant characteristics, and frequency of settling strategies. We then move on to investigating the impact of key covariates: infant sensory sensitivity, age, sleep onset latency, and sex on PIBBS settling behavior, using repeated measures ANOVA. Because of high correlations with infant sensory sensitivity, parental sensory sensitivity was not included in analysis. A follow‐up analysis asked whether the effects on sleep settling were due to differences in the number of different strategies used. A univariate ANOVA was run to test the impact of the same predictors (infant sensory sensitivity, age, sleep onset latency, and sex) on settling variability. Sleep onset latency was added as covariate in both ANOVAs to ascertain whether any associations between infant sensitivity and parental settling may be explained by how long infants took to fall asleep. All continuous variables were mean centered.

## 3 Results

### 3.1 Associations Between Key Variables

A bivariate correlation table with age partialled out (Table 2) shows that higher infant sensitivity is associated with longer sleep onset times (*r*(243) = 0.360, *p* < 0.01) and more frequent night awakenings (*r*(243) = 0.183, *p* < 0.01). Higher infant sensitivity correlates with more frequent use of all sleep strategy sub‐scales. Higher infant sensitivity strongly positively correlates with parental sensitivity (*r*(223) = 0.823, *p* < 0.01). The strong correlation between infant and parent sensory sensitivity (above the advised threshold of *r* = 0.7, Dormann et al. 2013) precludes using both infant and parental sensitivity in a model predicting parental settling strategies; the latter measure was therefore excluded from further analyses.

**TABLE 2:** Correlations between key variables, with age partialled out.

	Sleep onset latency	Awakenings	Infant sensory sensitivity	Active physical comforting	Movement	Social contact	Encourage autonomy	Give medicine	
Awakenings	**0.403**	—							
Infant sensory sensitivity	**0.360**	**0.178**	—						
Active physical comforting	**0.290**	**0.236**	**0.508**	—					
Movement	**0.190**	**0.139**	**0.533**	**0.444**	—				
Social contact	**0.333**	0.122	**0.719**	**0.589**	**0.540**	—			
Encourage autonomy	**0.212**	0.017	**0.648**	**0.236**	**0.383**	**0.632**	—		
Give medicine	0.109	−0.042	**0.704**	**0.395**	**0.428**	**0.570**	**0.572**	—	
Maternal sensory sensitivity	**0.361**	**0.193**	**0.825**	**0.417**	**0.358**	**0.605**	**0.597**	**0.594**	—

*Note:* Significant correlations (*p* < 0.05) highlighted in bold. Correlations between the 5 different scales of the Parental Interactive Bedtime Behavior Scale (PIBBS) highlighted in purple.

### 3.2 Associations Between Infant Sensitivity and Parental Settling Strategies

We ran a repeated‐measures ANOVA with settling types (Active physical comforting, Settle by movement, Social comforting, Encouraging autonomy, and Giving medication) as outcome variables and infant sensory sensitivity, age, sleep onset latency, and sex, as predictors. This model yielded a significant effect of settling type (F(3.48, 835.99) = 10.262, *p* < 0.001 Greenhouse‐Geisser correction for sphericity violation) with Active physical comforting being the most frequently used strategy and Give medicine, the least (Figure 1). Settling type also significantly interacted with infant sensitivity (F(3.48, 835.99) = 23.02, *p* < 0.001), and sleep onset latency (SOL) (F(3.48, 835.99) = 6.62, *p* < 0.001). There was also a significant main effect of infant sensitivity (F(1, 240) = 435.33, *p* < 0.001) but no significant main effect of SOL (F(1, 240) < 1) or age (F(1, 240) = 2.9, *p* = 0.09). While some variation in the association between sensitivity or SOL and different settling types is apparent, infant sensitivity is associated with higher use of all settling strategies (see Figure 1).

**FIGURE 1:**
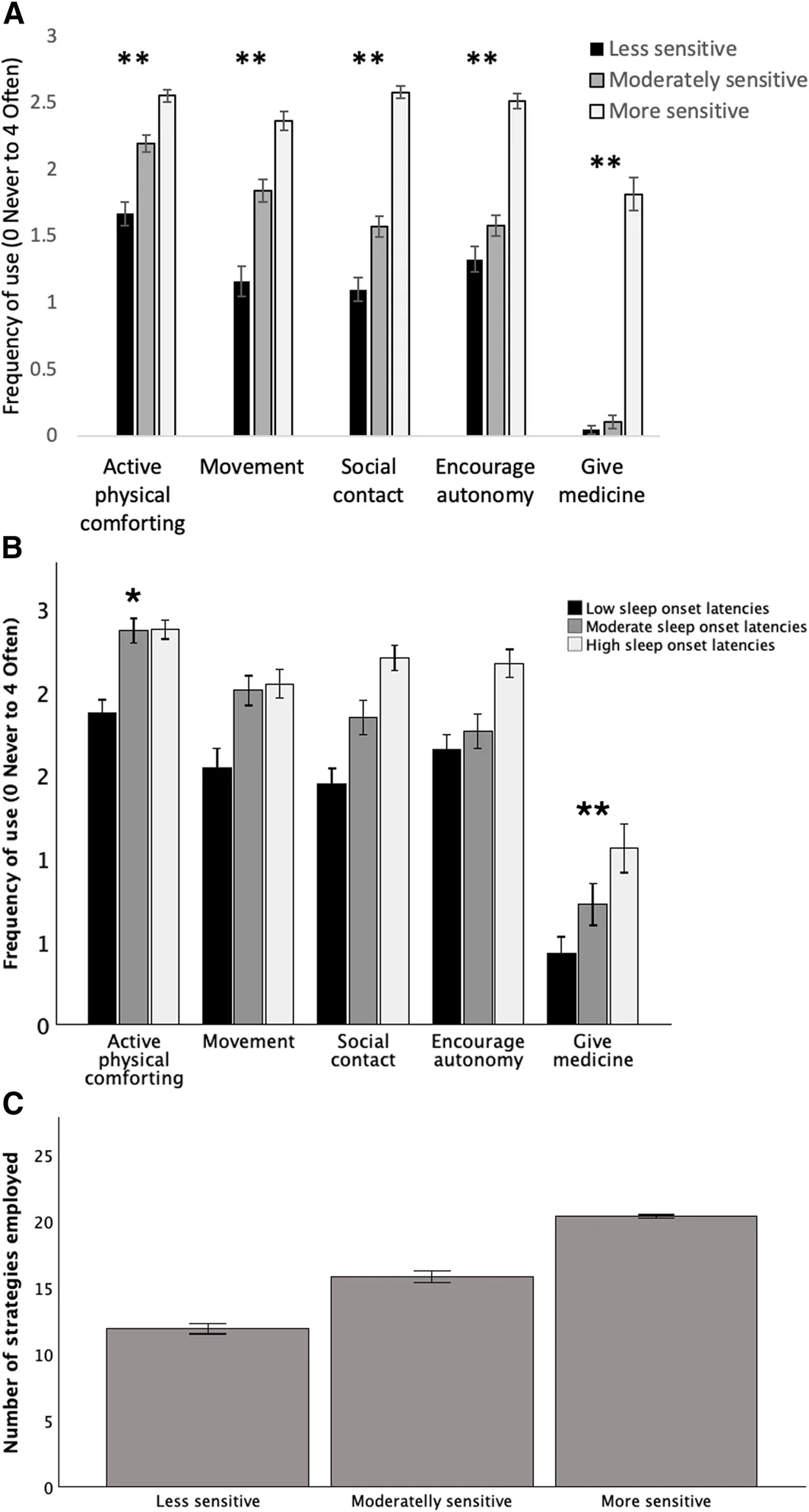
In the upper panel, parents of more sensitive infants employed all settling strategy types more frequently. In the middle panel, parents of infants with longer sleep onset latencies (SOL) employ some settling strategy types more frequently. In the lower panel, parents of more sensitive infants employed a larger number of different strategies. To depict the association between infant sensitivity or SOL and parental settling strategies, sensitivity and SOL categories were calculated using a tercile split (Low Threshold: Less sensitive < 1.61, *n* = 66; Moderately sensitive 1.61–2.88, *n* = 87; More sensitive > 2.88, *n* = 92; SOL: Low SOL < 15 min, *n* = 89; Moderate SOL 15–23.75, *n* = 72; High SOL > 23.75, *n* = 84). Error bars represent 1 SE. Post‐hoc univariate tests statistics are indicated by * *p* < 0.05, **p < 0.001.

### 3.3 Associations Between Infant Sensitivity and Settling Variability

Parents used between 2 and the maximum of 21 different settling strategies, with an average of 16. A univariate ANOVA, with the covariates of interest (sex, age, SOL and infant sensitivity), yielded only a main effect of infant sensitivity (F(1,245) = 231.94, *p* < 0.001), such that higher sensitivity was associated with a greater number of strategies used, as well as a main effect of SOL (F(1, 245) = 7.33, *p* = 0.007), such that the longer sleep onset latencies were, the higher the number of strategies employed.

## 4 Discussion

A growing body of literature suggests that increased sensory sensitivity may be a driver of poor sleep in development (De Laet et al. 2022; Appleyard et al. 2020). In this study, we confirm that an association between a general measure of sensory processing differences and sleep problems is present from very early in development, in infants under 14 months of age. Parents reported that more sensitive infants took longer to fall asleep and woke up more frequently during the night. Further, we found that parents used a variety of sleep settling strategies, ranging from more to less involved. Importantly, parents of more sensitive infants used more of these strategies, more frequently.

We took a transactional approach, to explore the broader connections between infant and parent characteristics and parental care around sleep. Based on existing literature, we tested two alternative hypotheses. The first hypothesis was that higher infant sensory sensitivity might lead to less frequent use of strategies rich in sensory input, such as Active physical comforting, as compared with strategies that encourage autonomy. The alternative hypothesis was that infant sensory hypersensitivity may lead to higher use of all strategies requiring parental involvement and a decrease in strategies encouraging autonomy. Our findings did not align with either hypothesis. Parents of more sensitive infants used all strategy types more often. Inspection of usage of all 21 strategies (Supporting Information S1: Figure S2) revealed that Active physical comforting strategies such as Stroking, Cuddling, and Feeding to sleep were highly used by all groups. In contrast, all other strategies were influenced by sensitivity, with some, such as Playing with the child, Leaving them to cry, and Giving medicine, strongly associated with infant sensitivity. This latter item is often removed from the PIBBS, because of low ratings, but is of clear relevance to a population with increased sleep problems and should be included in future studies.

Importantly, these findings emerged from models that accounted for infant sleep onset latencies. Longer sleep onset latencies were also associated with an increased used of certain settling strategy types, particularly with Active physical strategies and Giving medicine. Thus, while prolonged time spent settling infants was also related to more parental involvement, a direct effect of child sensitivity remains apparent. A follow‐up analysis of the number of different strategies used by parents also yielded significant associations with infant sensitivity and SOL, with parents of more sensitive infants or of those infants that took longer to fall asleep, using a larger number of strategies; this included sometimes incongruous strategies, for example, Playing with the child, or Leaving them to cry, indicative of increased effort and, potentially, increased stress, associated with settling to sleep.

The very strong association between infant and parental sensory sensitivity precluded us from using both these variables as predictors; we were therefore unable to test the extent to which parent versus child sensory sensitivity contributes to the choice of settling methods. The link between infant and parental sensitivity reports might result from shared genetic background, in line with strong estimates of heritability of sensory reactivity in children (Taylor et al. 2018). Parental sensitivity itself might associate with increased parental anxiety around sleep, something future studies should investigate. The role that parental sensory processes might play in parenting and caregiving has been understudied, but recent evidence suggests that maternal sensitivity is associated with a higher frequency of daily hassle (Gafni‐Lachter et al. 2022) and attachment insecurity (Branjerdporn et al. 2019). When strong correlations between variables preclude a quantitative approach to delineating their contributions, a different strategy may be helpful, such as using qualitative methods that inquire about the motivations behind settling decisions. For example, Turner et al. (2012) captured the parenting struggles of four mothers with sensory processing challenges; although the focus was not on sleep, mothers in this study mentioned that bedtime routines were particularly challenging because of the physical proximity involved.

Results of our study should be considered in light of several limitations. Data were collected during Covid‐19 pandemic (in 2021), which might have impacted both infant sleep and parental mental health. However, another large study conducted during Covid‐19 UK lockdown found that pre‐school children were minimally affected, with many sleeping better than they had before lockdown (Gliga et al. 2023). Another limitation is the use of parent‐report measures of infant sleep, which may have low reliability. Camerota et al. (2018) found good agreement between parent report, actigraphy, and video‐somnography when measuring 3‐month‐olds’ sleep schedule variables (like sleep onset and wake time); in this study there was only moderate agreement for sleep fragmentation variables (including night awakenings and the longest sleep period). Finally, we only collected data on parent sensitivity from a subset of the participants (82%); this was due to adding this questionnaire to the study at a later point, rather than certain parents choosing not to complete it.

A growing body of work now substantiates a link between the quality of early sleep and later cognitive development (e.g., Begum‐Ali et al., 2022) as well as early disruptions in sleep in neurodevelopmental conditions (e.g., De Laet et al. 2022). This work highlights the importance of intervening to promote sleep, from as early as possible in infancy. However, research on early sleep interventions rarely consider sources of these disturbances. Our work suggests that, where sleep difficulties exist, they may be associated with broader familial factors that act as stressors, such as infant and parent sensory traits. Our findings highlight the importance of using sensory sensitivity as a stratifying dimension when investigating sleep difficulties in typical and atypical development. Future research should measure parental mental health more broadly (e.g., anxiety, depression) as well as the process of decision‐making around sleep, to understand the mechanistic pathways linking sensory sensitivity and sleep.

In conclusion, we show an association between sensory sensitivity and sleep in young infants. We find that parents use a wide variety of settling strategies, and that parents of infants with sensory sensitivities use more strategies, with higher frequency. Our findings highlight the importance of a broader approach to understanding infant sleep, that considers factors intrinsic to the child and their relation to nurturing care. We hope our findings will motivate future studies looking at how settling strategies interact with sensitivity profiles to modulate infant sleep and development.

## Author Contributions

**Teodora Gliga:** conceptualization, funding acquisition, investigation, writing – original draft, writing – review and editing, visualization, validation, methodology, project administration, formal analysis, software, resources, supervision, data curation. **Hope Fincham:** investigation, methodology, writing – original draft, writing – review and editing. **Anna De Laet:** data curation, investigation, validation, visualization, writing – review and editing. **Rachael Bedford:** conceptualization, investigation, writing – original draft, writing – review and editing, methodology, supervision.

## Ethics Statement

The study was granted ethical approval by the University of East Anglia on 10th March 2021.

## Supporting information


Supporting Information S1

## Data Availability

The data that support the findings of this study are available from the corresponding author upon reasonable request.
