# Supplementary material for: Sensory Hyper‐Sensitivity Associates With Poor Infant Sleep and Different Parental Sleep Practices, in a UK Cohort
Source: Infancy. 2026 Oct 7;31(5):e70129. doi: 10.1111/infa.70129 (PMC13646340; doi:10.1111/infa.70129)
Supplement: Supplementary file 1 [file INFA-31-0-s001.docx]

**Supplementary Online Material**

Figure S1. Age and family income distributions. The median household income in the UK was approximately £32.5k in 2023

<https://www.gov.uk/government/statistics/households-below-average-income-for-financial-years-ending-1995-to-2023/households-below-average-income-an-analysis-of-the-uk-income-distribution-fye-1995-to-fye-2023>

Figure S2 Usage of various settling strategies by parents of less, moderately and more sensory sensitive infants.

*Table S1. Demographic characteristic separated by whether parents reported an older sibling with autism or no older sibling with autism.*

|  | **With** older autistic sibling | **Without** older autistic sibling |
| --- | --- | --- |
| Age (months)  Range  N | 8.25  6-13  44 | 8.14  6-13  201 |
| Sex (Boy:Girl)  N | 32:15  44 | 123:106  201 |
| SOL (min)  Range  N | 22.29  5-70  44 | 22.28  0-77.5  201 |
| Awakenings  Range  N | 2.95  1-6  47 | 2.68  0-14  201 |
| Infant sensitivity  Range  N | 2.89  0.6-4.1  44 | 2.19  0-4.16  201 |
| Maternal GSQ  Range  N | 79.70  12-115  44 | 61.24  3-123  181 |
|  |  |  |

Figure S3. Differences in the distribution of sensory sensitivity in infants whose parents reported having or not having an older autistic child.
